# Cooperation of immune regulators Tollip and surfactant protein A inhibits influenza A virus infection in mice

**DOI:** 10.1186/s12931-024-02820-3

**Published:** 2024-05-03

**Authors:** Niccolette Schaunaman, Diana Cervantes, Taylor Nichols, Mari Numata, Julie G. Ledford, Monica Kraft, Hong Wei Chu

**Affiliations:** 1 Department of Medicine, National Jewish Health, 1400 Jackson Street, Room A639, Denver, CO 80206 USA; 2 University of Arizona, Tucson, AZ USA; 3 Icahn School of Medicine at Mount Sinai, New York, NY USA

**Keywords:** Tollip, Surfactant protein A, Influenza A virus, Neutrophilic inflammation

## Abstract

**Background:**

Influenza A virus (IAV) infection is a significant risk factor for respiratory diseases, but the host defense mechanisms against IAV remain to be defined. Immune regulators such as surfactant protein A (SP-A) and Toll-interacting protein (Tollip) have been shown to be involved in IAV infection, but whether SP-A and Tollip cooperate in more effective host defense against IAV infection has not been investigated.

**Methods:**

Wild-type (WT), Tollip knockout (KO), SP-A KO, and Tollip/SP-A double KO (dKO) mice were infected with IAV for four days. Lung macrophages were isolated for bulk RNA sequencing. Precision-cut lung slices (PCLS) from WT and dKO mice were pre-treated with SP-A and then infected with IAV for 48 h.

**Results:**

Viral load was significantly increased in bronchoalveolar lavage (BAL) fluid of dKO mice compared to all other strains of mice. dKO mice had significantly less recruitment of neutrophils into the lung compared to Tollip KO mice. SP-A treatment of PCLS enhanced expression of TNF and reduced viral load in dKO mouse lung tissue. Pathway analysis of bulk RNA sequencing data suggests that macrophages from IAV-infected dKO mice reduced expression of genes involved in neutrophil recruitment, IL-17 signaling, and Toll-like receptor signaling.

**Conclusions:**

Our data suggests that both Tollip and SP-A are essential for the lung to exert more effective innate defense against IAV infection.

**Supplementary Information:**

The online version contains supplementary material available at 10.1186/s12931-024-02820-3.

## Background

Influenza A virus (IAV), specifically H1N1, is a respiratory virus commonly known as the flu that causes seasonal epidemics, but most importantly was responsible for the 2009 pandemic [1]. IAV remains a significant risk factor for viral pneumonia and acute exacerbations for respiratory diseases including asthma [2]. The dysfunction of innate immune responses has been proposed to contribute to the severity of infection [3, 4], but the exact mechanisms are still unclear.

Toll-interacting protein (Tollip) is an innate immune regulator that is expressed in epithelial cells, macrophages, alveolar type II cells, and basal cells [5–9]. Tollip has been found to play a wide role in regulating cellular processes such as autophagy, mitochondrial function, and innate immunity. Our group has found that Tollip is important in promoting an anti-viral response during different viral infections [7, 10, 11]. We found that Tollip inhibits excessive inflammation induced by rhinovirus by inhibiting the activation of IRAK-1 and by promoting the production of soluble ST2, a decoy receptor for IL-33. Tollip is also able to inhibit IAV-induced inflammation through the inhibition of extracellular ATP and IL-33 release. We also demonstrated that Tollip single nucleotide polymorphism (SNP) rs5743899 was correlated with significantly less Tollip expression in humans, lower pulmonary function in asthmatics, and impaired anti-viral responses following rhinovirus infection. Tollip as an adaptor protein can interact and cooperate with other intracellular proteins including STAT3, IRAK1, TLR2/4, and IL1R1 [8, 12–15]. While our group has studied Tollip during viral infections, we have not delved into how Tollip cooperates with other host defense proteins such as surfactant protein A (SP-A) to induce an effective immune response, specifically during IAV infection.

SP-A, one of four surfactant proteins produced and secreted primarily from lung alveolar type II cells, has multiple functions in lung immunity. SP-A is a hydrophilic protein including an N-terminal triple-helical collagen region that interacts with immune cells, and a carbohydrate recognition domain (CRD) that acts as a pattern recognition receptor by recognizing proteins on the outside of microbes and allergens [16]. It serves as a first line of defense against inhaled pathogens [17–19]. Our group found that SP-A acts as an opsonin by binding to the proteins and lipids found on *Mycoplasma pneumoniae* reducing its growth and pathogenicity [20]. SP-A can also bind to the glycoproteins found on the envelope of IAV, resulting in neutralization and/or the enhanced phagocytosis of the viruses by surrounding macrophages [18, 21]. SP-A has also been shown to reduce inflammation present in asthmatic patients by reducing phosphorylation of STAT3, an important intermediate for type 2 inflammation. SP-A expression has been found to be decreased in patients with asthma, and several SNPs within SP-A may be responsible for decreased ability to bind to virus leading to worsened infection [22, 23]. In vivo, the role of SP-A during IAV infection remains controversial depending on the dose of infection and time post infection when mice were sacrificed. Levine et al. found that SP-A knockout (KO) mice had significantly more viral load in lung tissue and enhanced neutrophil infiltration compared to wild-type (WT) mice at three and five days post infection [24], while another study conducted by Li et al. found that there was no difference in viral load between SP-A KO mice and WT mice two days and six days post infection [25]. Interestingly, SP-A can modulate inflammation through the interaction with other innate molecules, such as CD91, SP-R210, and Toll-like receptor 4 (TLR4) [26].

While it has been shown that Tollip and SP-A modulate inflammation separately, their cooperation in lung defense against viral infection remains unknown. In the present study, we hypothesized that Tollip and SP-A cooperate to more effectively defend against IAV infection. To test this hypothesis, we performed an acute IAV infection experiment using our newly generated Tollip/SP-A double knockout (dKO) mice, and in precision-cut lung slice (PCLS) model which maintains the three-dimensional structure of the lung and the interactions of multiple cell types (e.g., alveolar epithelial cells, monocytes, and macrophages).

## Methods

### Influenza A virus (IAV) preparation

The virus used in this study was Pandemic Influenza A/California/04/2009 (CA04) virus, which was kindly provided by Dr. Kevin Harrod from University of Alabama at Birmingham. The virus was propagated in Madin-Darby canine kidney (CCL-34, MDCK, ATCC, Manassas, VA) cells, as previously published [27–31], and tittered by quantitative plaque assay using MDCK cells [29].

### Mice

Tollip knockout (KO) mice on a C57/BL6 background were obtained from Dr. Liwu Li [32] at Virginia Polytechnic Institute and State University and bred at the National Jewish Health (NJH) Biological Resource Center (BRC). Surfactant Protein A (SP-A) KO mice on a C57/BL6 background were obtained from Dr. Julie Ledford [33, 34] at the University of Arizona and then bred at the BRC of NJH. Tollip/SP-A double KO (dKO) mice were generated at NJH by crossing Tollip KO and SP-A KO strains, and then inbred for at least six generations to produce Tollip/SP-A dKO mice. Wild-type (WT) C57/BL6 mice were purchased from the Jackson Laboratory (Bar Harbor, ME), and housed with the KO mice at NJH BRC under pathogen-free housing conditions. All the experimental protocols were approved by the Institutional Animal Care and use Committee at NJH.

### Genotyping of Tollip/SP-A dKO mice

To confirm Tollip/SP-A double knockout, lung tissue from each mouse was digested for DNA extraction using the REDExtract-N-Amp reagent kit (XNAT-100RXN, MilliporeSigma, Burlington, MA) following the manufacturer’s instructions. The DNA product underwent PCR using custom primers (Table 1) [13, 34] for genotyping.

**Table 1:** Custom primer sequences for Tollip/SP-A genotyping

Primer	Sequence
Tollip Sufficient Forward Primer	5’-GGATTTGGGATTCATCAGAGGC-3’
Tollip Sufficient Reverse Primer	5’-ACAAGAGTGGGACGGAAAACTTC-3’
Tollip Deficient Reverse Primer	5’-GGAGAGGCTATTCGGCTATG-3’
SP-A Sufficient Forward Primer	5’-ACAGAAGTTTGTGCCGGAAG-3’
SP-A Sufficient Reverse Primer	5’-ATGGTCACCCAGAAAACAGG-3’
SP-A Deficient Reverse Primer	5’-GCTACTTCCATTTGTCACGTCC-3’

The PCR product was run on a 1% agarose gel, and the band size determined the genotypes. Tollip sufficient mice had a single band around 600 base pairs (bp), Tollip deficient mice had a single band approximately 1,100 bp (Supplementary Fig. 1A), which includes a neomycin cassette. SP-A sufficient mice had a single band at 167 bp, homozygous SP-A KO mice had a single band at 320 bp (Supplementary Fig. 1B), which includes a pGKneoBPA insert.

### Mouse model of IAV infection

**Fig. 1:**
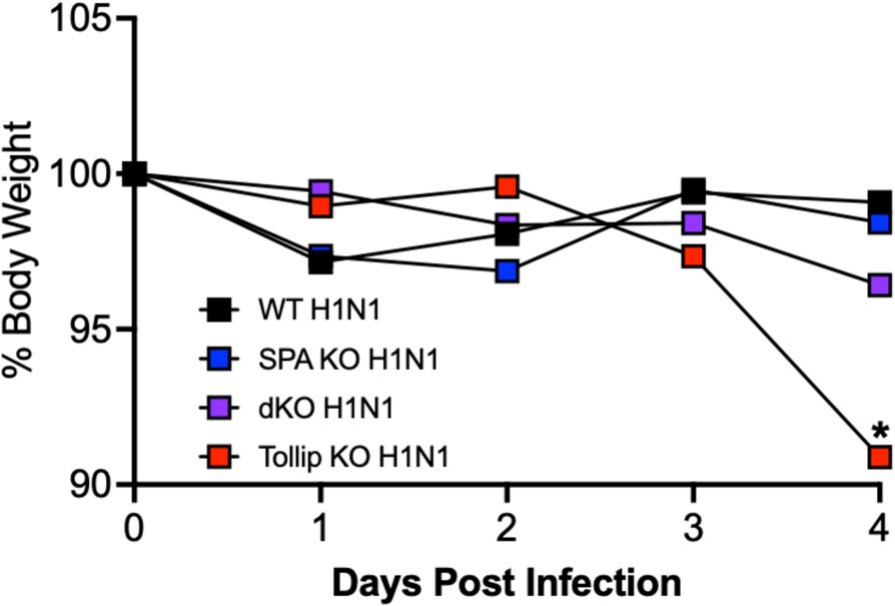
Body weight measurements during four days of IAV infection. Tollip KO mice have significantly more weight loss than all other strains. Interestingly, dKO mice lost weight after IAV infection, but there was no statistical significance between dKO mice and WT and SP-A KO mice

dKO, Tollip KO, SP-A KO, and WT mice, ages 8–12 weeks (age and gender matched), were inoculated intranasally with 1 × 10^2^ PFU/mouse of IAV or 50 µl of PBS as a control. We have previously published IAV infection mouse models where Tollip deficient (vs. wild-type) mice sacrificed after four, seven, and ten days of infection consistently demonstrated more viruses [7, 35]. We focused on the earlier time point (i.e., four days post infection) for this study, because SP-A is one of the first lines of host defense in the lung to interact with inhaled pathogens. Since a low dose of IAV was used, mice survived through the entire course of viral infection (four to ten days). For the current four-day study, we measured body weight over the course of the experiment (Fig. 1).

### Mouse bronchoalveolar lavage (BAL) fluid and lung tissue processing

Mice were euthanized by intraperitoneal injection of pentobarbital sodium (Fatal-Plus) in sodium chloride. Lungs were lavaged with 1 ml of sterile saline. Cell-free BAL fluid was used for viral load measurement and cytokine measurement. BAL fluid cell differential cytospin slides were stained with a Diff-Quick stain kit (23–122929, 23–122937, 23–122952, Fisher Scientific, Hampton, NH) for cell differential counts. Leukocyte differentials were determined as a percentage of 500 counted leukocytes. The left lung was used for viral load and antiviral mRNA expression, and the right lungs were used for macrophage isolation.

### Isolation of macrophages from mouse lung tissue

Right lung tissues from IAV infected WT, Tollip KO, SP-A KO, and dKO mice were digested with proteases and DNase as previously published [36]. After digestion, the tissues were passed through a 200 µm strainer followed by a 40 µm cell strainer and centrifuged to collect the cells. Red blood cells were lysed, and the remaining cells were resuspended in DMEM with 10% fetal bovine serum (FBS, F31016-500, SeraPrime LLC, Fort Collins, CO) and penicillin/streptomycin (15140122, ThermoFisher, Waltham, MA) and plated in a 24-well plate at 37°C, 5% CO_2_. Macrophages were isolated by adherence for at least two hours, after which adhered cells were washed with PBS and saved in RLT Lysis Buffer (79216, Qiagen, Hilden, Germany) for RNA extraction. We chose to isolate macrophages over other cell types as lung macrophages have been shown to protect against IAV infection [37].

### Mouse precision-cut lung Slice (PCLS) culture and infection

Naïve WT, Tollip KO, SP-A KO, and dKO mouse lungs were inflated with 1.5% low-melting agarose (BMA50002, Fisher Scientific, Hampton, NH) and sliced into consecutive 250 µm thick sections using a Compresstome VF-300 vibratome (Precisionary Instruments, Natick, MA). The slices were transferred into a 24-well plate containing DMEM media with antifungal agents and antibiotics and incubated overnight at 37˚C supplemented with 5% CO_2_. Slices were then pre-treated with 10 µg/ml of recombinant murine SP-A (abx166071, Abbexa Ltd, Cambridge, United Kingdom) for 30 min, and were thereafter infected with 3 × 10^5^ PFU/well of IAV for two hours at 37˚C. Slices were then washed three times with PBS to remove the unbound virus, and fresh DMEM with 10 µg/ml of recombinant murine SP-A was added back to the slices. Supernatants and tissue were harvested 48 h post infection.

To determine if SP-A inhibits viral entry or viral replication, naïve WT mouse lungs were inflated and sliced as stated above. Slices were pre-treated with 10 µg/ml of recombinant murine SP-A for 30 min, and were then infected with 3 × 10^5^ PFU/well of IAV for two hours at 37˚C. Slices were then washed three time; the first with PBS, the second with PBS containing 1 mg/ml proteinase K to remove viral particles that may be attached to the cell surface but had not entered the cells [38], and the third time with PBS. Slices were then harvested immediately (time 0 h). Fresh DMEM with 10 µg/ml of recombinant murine SP-A was added back to the remaining slices which were then harvested 24 h, and 48 h post infection.

### Reverse transcription and quantitative real-time (RT-qPCR)

Intracellular and released IAV was measured by RT-qPCR. Intracellular IAV was measured from homogenized lung tissue, and RNA was isolated using the TRIzol reagent method. Released IAV was measured from BAL fluid of infected mice, where equal volumes of BAL fluid were used to extract RNA using Mini Spin Columns (1940–250, Epoch Life Science Inc., Missouri City, TX) according to the manufacturer’s instructions.

Total RNA was extracted from mouse PCLS as we previously described [39]. Briefly, PCLS tissue was homogenized using the TRIzol reagent (15596018, ThermoFisher, Waltham, MA), followed by using Mini Spin Columns for RNA extraction (1940–250, Epoch Life Science Inc., Missouri City, TX) according to the manufacturer’s instructions.

Custom-made primers and probe (Integrated DNA Technologies, Coralville, IA) for the M1 gene of IAV were 5’-GACCRATCCTGTCACCTCTGAC-3’ (forward), 5’-AGGGCATTYTGG-ACAAAKC-3’ (reverse), and 5’-TGCAGTCCTCGCTCACTGGGCACG-3’ (probe) [40], and IFN-beta were 5’-GACGGAGAAGATGCAGAAGAG-3’ (forward), 5’-CCACCCAGTGCTGGAGAA-3’ (reverse), and 5’-TGCCTTTGCCATCCAAGAGAT-3’ (probe). TaqMan Gene Expression Assay (ThermoFisher, Waltham, MA) was used to determine relative mRNA levels of NOS2 (Mm00440502) and TNF-α (Mm00443258).

Target gene expression was normalized to the housekeeping gene 18S rRNA (ThermoFisher, Waltham, MA). The comparative threshold cycle method (∆∆Ct) was applied to determine the relative levels of target genes.

### Plaque assay

Serial dilutions of BAL fluid and half of the left lung homogenate from IAV-infected mice were plated on MDCK cells for one hour at 37˚C. Viral titer was calculated by quantitative plaque assay as described previously [29].

### ELISAs

LIX, also known as CXCL5, was measured in BAL fluid supernatants using a Mouse LIX DuoSet ELISA kit (DY443, R&D Systems, Minneapolis, MN) following manufacturer’s instructions.

### Bulk RNA sequencing and analysis

RNA extracted from isolated lung macrophages was used for bulk RNA sequencing as described previously [36]. Briefly, preparation of RNA library, transcriptome sequencing, and downstream analysis were conducted by Novogene Co., LTD (Beijing, China). The RNA library was generated by using the NEBNext Ultra II RNA library prep kit (New England BioLabs Inc., Ipswich, MA) with a poly-A mRNA selective workflow (non-directional, no rRNA depletion step). RNA samples were then sequenced using Illumina PE150 technology with a target output of 6 Gb PE150 data (20 M PE150 read-pairs/40 M individual 150 bp reads). ClusterProfiler R package was utilized by Novogene Co., LTD to test the statistical enrichment of differential expressed genes in KEGG pathways. Additionally, genes with a *p*-value < 0.05 and log2-fold change > 0 between the groups were assigned as differentially expressed and were included for pathway analysis using the functional enrichment analysis of the Database for Annotation, Visualization and Integrated Discovery (DAVID) Bioinformatic Resources [41, 42], which was developed by the Laboratory of Human Retrovirology and Immunoinformatics (LHRI) in collaboration with the National Institute of Allergy and Infectious Diseases (NIAID).

To determine the transcriptomic changes of the entire macrophage population, lung macrophages were isolated for bulk RNA sequencing. It has been shown that macrophages present in lung tissue are largely comprised of alveolar macrophages and interstitial lung macrophages [43–45]. Our RNA sequencing data clearly showed the markers for alveolar macrophages including SiglecF, CD68, and MARCO. Raw Bulk RNA sequencing data are provided as additional files (Additional files 1, 2, 3, 4, 5, 6, 7, 8, 9 and 10).

### Statistical analysis

Statistical analyses were performed with GraphPad Prism 10 software, and graphical data were presented as median with interquartile range (IQR). Due to data variation in our models, all data were analyzed as nonparametric data using the Mann–Whitney U test for two group comparisons, and Kruskal–Wallis test followed by Dunn’s test for multiple comparisons. A *p*-value < 0.05 was considered statistically significant.

## Results

### Tollip/SP-A double knockout (dKO) mice demonstrate increased lung viral load

**Fig. 2:**
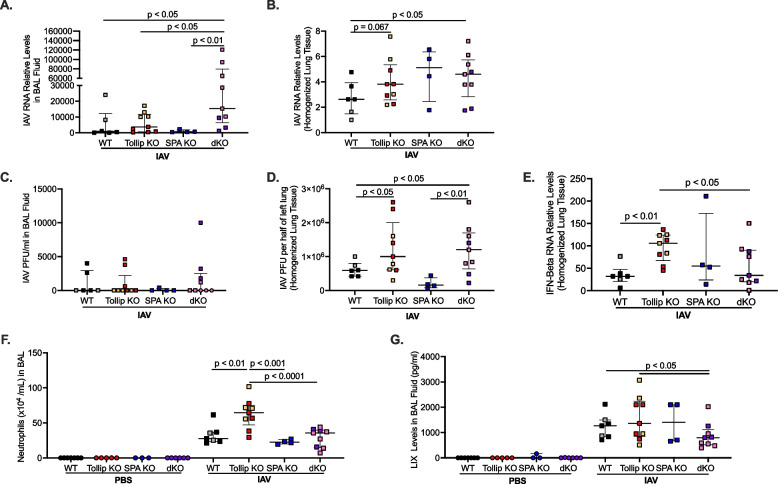
SP-A/Tollip double knockout increased viral load but did not induce a robust inflammatory response. Wild-type (WT), Tollip KO, SP-A KO, and Tollip/SP-A double KO (dKO) mice were intranasally infected with 1 × 10^2^ PFU/mouse of IAV or PBS for four days. After four days of IAV infection dKO mice had significantly more IAV mRNA levels present in (**A**) BAL fluid and (**B**) homogenized lung tissue. These data were recapitulated in plaque forming units of IAV found in (**C**) BAL fluid and (**D**) homogenized lung tissue. Tollip KO mice had significantly more IFN-beta mRNA expression compared to WT and dKO mice (**E**). dKO had significantly less neutrophils (**F**) and (**G**) LIX compared to Tollip KO mice. Each symbol represents an individual mouse (*n* = 4–9 mice per group) and the different colored symbols represent an individual/independent experiment where a different batch of IAV was used (*n* = 2 total individual/independent experiments)

Viral RNA levels were measured in BAL fluid of infected mice (Fig. 2A) four days after IAV infection. While SP-A KO mice had similar levels of viral load in BAL fluid compared to WT mice, dKO mice had significantly more virus released into the BAL fluid compared to all three strains of mice. This suggests that SP-A deficiency alone is not able to worsen viral infection in Tollip sufficient mice. Viral RNA levels measured in homogenized lung tissue of infected mice (Fig. 2B) were also significantly higher in dKO mice compared to WT mice, but there was not a statistically significant difference compared to Tollip KO and SP-A KO mice. Tollip KO mice and SP-A KO mice trended to have more viral RNA in lung tissue compared to WT mice, but this was also not statistically significant. Additionally, plaque assay was performed to quantify levels of infectious viruses. Unlike the viral RNA data, IAV PFU level in the BAL fluid was not different among various groups of mice as PFU was undetectable in some mice (Fig. 2C). Our plaque assay data in BAL fluid could be explained by the fact that BAL fluid may contain antiviral components that inactivate or lessen the infectivity of IAV and other respiratory viruses [46–48]. PFU data in the homogenized lung showed similar results to lung tissue viral RNA levels where dKO mice had significantly higher levels of PFU than WT mice and SP-A KO mice. Tollip KO mice had significantly higher levels than WT mice (Fig. 2D). This data along with the BAL viral load, suggests that dKO mice had an impaired ability in clearing the viruses.

Tollip KO mice infected with IAV were able to significantly increase IFN-beta mRNA expression compared to WT and dKO mice (Fig. 2E). There was no significant difference in IFN-beta levels in WT, SP-A KO, and dKO mice.

### Tollip/SP-A dKO mice lack a robust antiviral inflammatory response after IAV infection

After IAV infection, there was a significant increase in neutrophil numbers in the BAL fluid of all strains of mice (Fig. 2F). Tollip KO mice infected with IAV had significantly more neutrophils than WT mice. SPA KO mice had similar number of neutrophils as compared to the WT mice. In contrast to the viral load data in BAL fluid, dKO mice had significantly lower numbers of neutrophils than the Tollip KO mice and failed to increase neutrophils as compared to the WT mice. Neutrophils have been shown to be able to limit viral infection in mice [49, 50].

LIX (CXCL5), a neutrophil chemokine, was significantly upregulated in all strains of mice after four days of IAV infection (Fig. 2G). dKO mice had significantly less LIX release compared to WT and Tollip KO mice. These data suggest that dKO mice have impaired ability to initiate a neutrophil recruitment process.

### Exogenous SP-A treatment reduces viral load in Tollip/SP-A deficient precision-cut lung slices (PCLS)

**Fig. 3:**
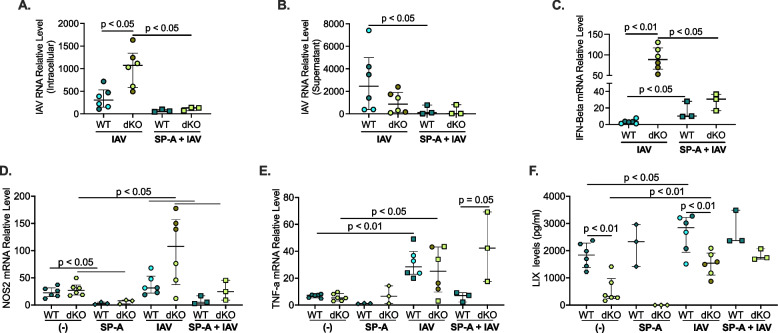
Recombinant SP-A treatment reduces viral load in Tollip/SP-A deficient mouse precision-cut lung slices (PCLS). PCLS from naïve WT and dKO mice were pre-treated with 10 µg/ml of recombinant mouse SP-A and then infected with 3 × 10^5^ PFU/well of IAV for 48 h. **A** dKO had significantly more viral load in PCLS tissue, which was significantly decreased by SP-A. **B** SP-A decreased viral load released into the supernatant of WT and dKO slices. **C** dKO PCLS had significantly higher IFN-beta mRNA expression levels compared to WT. SP-A increased IFN-beta in WT PCLS. **D** IAV increased NOS2 levels in dKO slices. IAV-infected dKO slices trended to have higher NOS2 mRNA levels than WT slices. SP-A treatment reduced NOS2 in WT and dKO slices with and without infection. **E** IAV infection significantly increased TNF mRNA levels in both WT and dKO slices. TNF levels were similar between WT and dKO PCLS after infection, but SP-A treatment trended to increase TNF levels in dKO slices. **F** LIX release was significantly less in dKO slices without infection. IAV infection significantly increased LIX release in both strains, but IAV-infected dKO slices had significantly less LIX release than WT. SP-A treatment did not increase LIX levels. Each symbol represents an individual PCLS slice from *n* = 1 mouse and the different colored symbols in the IAV group represent an individual/independent experiment (*n* = 2 total individual/independent experiments)

To determine if SP-A restores the antiviral function in lung tissue with both SP-A and Tollip deficiency, we treated PCLS from naïve WT and dKO mice with recombinant SP-A, followed by IAV infection for 48 h. As shown in Fig. 3A, dKO lung tissue had significantly higher tissue viral load compared to WT slices. With the addition of recombinant SP-A, dKO lung tissue showed a significant decrease in viral load. SP-A significantly reduced viral levels in supernatants of PCLS of WT mice, but not dKO mice (Fig. 3B).

To further understand the role of SP-A during IAV infection in PCLS, we measured anti-viral genes Interferon-beta (IFN-beta), and macrophage activation-associated cytokine TNF-α. IFN-beta RNA expression was significantly higher in dKO slices compared to WT slices (Fig. 3C). The addition of recombinant SP-A significantly increased IAV-induced IFN-beta levels in WT slices, and decreased IFN-beta levels in dKO slices to levels similar to WT treated with SP-A. As nitric oxide (NO) may be involved in IAV infection [51–53], we measured NOS2 mRNA expression. We found that IAV infection significantly increased NOS2 mRNA expression in dKO slices, but no in WT slices. dKO PCLS infected with IAV trends to have more NOS2 mRNA expression than IAV-infected WT PCLS (Fig. 3D). SP-A treatment significantly reduced NOS2 mRNA expression both with and without IAV infection in both WT and dKO mice. In the infected lung slices this was consistent with the intracellular IAV load and IFN-beta levels. TNF-α mRNA expression was measured (Fig. 3E). IAV infection significantly increased TNF-α mRNA levels in both WT and dKO slices. There was no difference of TNF-α expression between WT and dKO PCLS infected with IAV alone. However, in SP-A-treated PCLS with IAV infection, dKO tissue had higher levels of TNF-α mRNA expression than WT tissue. LIX protein was measured in supernatants, and at baseline dKO lung slices had significantly less LIX released than WT lung slices. IAV infection significantly increased LIX levels in WT and dKO lung slices, and like the in vivo model, dKO slices infected with IAV had significantly less LIX than WT lung slices. Interestingly, SPA treatment did not increase LIX levels (Fig. 3F). All these data suggest that exogenous SP-A treatment reduced viral levels, which was associated with a decrease in IFN-beta and NOS2 mRNA expression and increased TNF expression in dKO lung tissue.

**Fig. 4:**
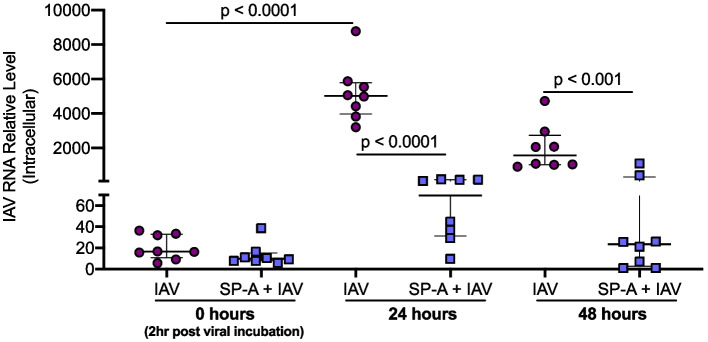
Recombinant SP-A treatment inhibits IAV replication in WT mouse precision-cut lung slices (PCLS). PCLS from naïve WT mice were pre-treated with 10 µg/ml of recombinant mouse SP-A and then infected with 3 × 10^5^ PFU/well of IAV for 0, 24, and 48 h. There was a significant increase in IAV load 24 h post infection compared to 0 h of infection. SP-A treatment did not change viral load at 0 h, however, SP-A significantly decreased IAV load 24 and 48 h post infection. Each symbol represents an individual PCLS slice from *n* = 1 mouse done in one individual/independent experiment

The role of SP-A during pathogen infections has been attributed to its direct binding to the virus particle to aid in phagocytosis [54]. To further determine how SP-A inhibits viral infection, we measured viral load in WT PCLS treated with and without recombinant SP-A at time of virus removal (0 h) and 24- and 48-h post infection (Fig. 4). At 0 h there is no difference in viral load with and without SP-A treatment. This suggests that SP-A may not inhibit viral entry. After 24 h of infection, there was a significant increase in IAV load compared to 0 h, confirming that IAV replicated in PCLS. Interestingly, SP-A treatment significantly reduced viral load at 24 and 48 h compared to virus alone. This set of data suggests that SP-A may inhibit viral replication.

### Impaired antiviral inflammatory gene expression in Tollip/SP-A deficient macrophages

To elucidate the potential mechanism whereby Tollip/SP-A deficiency impaired the antiviral function, we performed bulk RNA-sequencing in lung macrophages isolated from WT, Tollip KO, SP-A KO, and dKO mice with or without IAV infection.

**Fig. 5:**
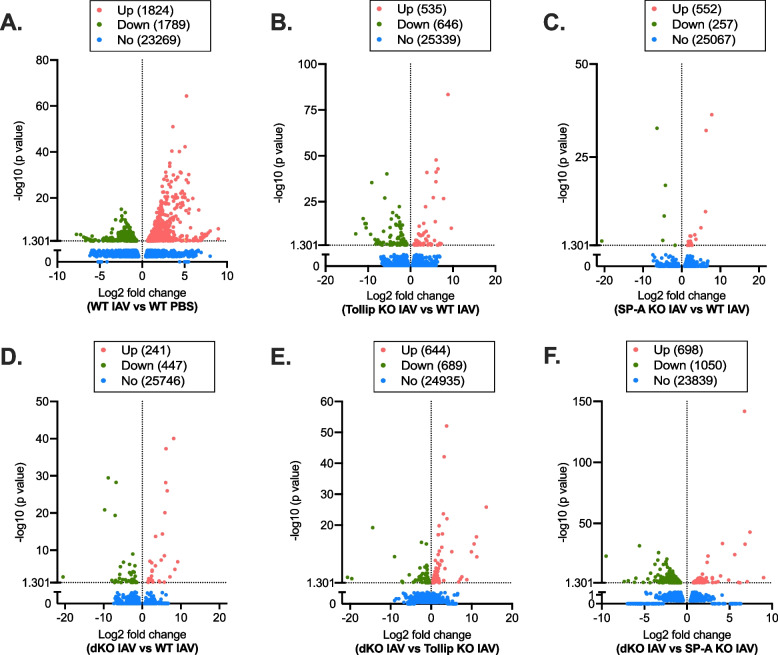
Volcano plots of differentially regulated genes in isolated lung macrophages from IAV-infected mice. **A** Genes up- and down-regulated in IAV-infected WT macrophages compared to PBS treated (wild-type, WT) macrophages. **B** Genes up- and down- regulated in IAV-infected Tollip KO (vs. WT) macrophages. **C** SP-A KO and **D** dKO macrophages compared with WT macrophages infected with IAV. **E** Genes up- and down- regulated in IAV-infected dKO macrophages compared to IAV infected Tollip KO macrophages and **F** IAV infected SP-A KO macrophages

In WT macrophages, IAV infection as compared to PBS control, up-regulated 1,824 genes while it down-regulated 1,789 genes (Fig. 5A). As expected, IAV infection, as compared to the PBS control in WT macrophages increased genes associated with pathways such as IL-17 signaling, TNF signaling, RIG-I-like receptor signaling, Toll-like receptor signaling, and NF-kappa B signaling (Supplementary Tables 1 and 2), and down-regulated pathways such as focal adhesion and tight junctions.

IAV infected Tollip KO macrophages, as compared to IAV infected WT macrophages, up-regulated 535 genes and down-regulated 646 genes (Fig. 5B). Pathways associated with the up-regulated genes were natural killer cell mediated cytotoxicity and cell adhesion molecules. The down-regulated genes were associated with pathways such as HIF-1 signaling and MAPK signaling (Tables 2 and 3).

**Table 2:** Pathways of genes altered by Tollip deficiency (vs. Tollip/SP-A sufficiency) in mouse lung macrophages infected with IAV

Signaling Pathways	Gene Count	%	Up- or Down- Regulated	*P* value
Natural killer cell mediated cytotoxicity	10	6.5	Up	1.90E-04
Cell adhesion molecules (CAMs)	8	5.2	Up	1.76E-02
HIF-1 signaling pathway	11	6.1	Down	9.59E-05
MAPK signaling pathway	13	7.2	Down	4.76E-02

**Table 3:** List of genes in selected pathways altered by Tollip deficiency (vs. Tollip/SP-A sufficiency) in mouse lung macrophages infected with IAV

**Up-regulated genes by Tollip deficiency vs. Tollip/SP-A sufficiency**			
Genes	Log2 fold change	Genes	Log2 fold change
Natural killer cell mediated cytotoxicity		Cell adhesion molecules (CAMs)	
Klrc1	1.18	H2-M2	3.69
Gzmb	1.17	Ctla4	1.25
Fasl	1.35	Tigit	0.93
Klrc2	1.21	Icos	1.24
Prf1	0.77	Cd28	1.03
Klra4	0.78	Pdcd1	0.89
Ncr1	1.18	Lrrc4b	6.34
Fcgr4	0.87	Itga8	1.56
Klrb1c	0.68		
Ulbp1	0.66		
**Down-regulated genes by Tollip deficiency vs. Tollip/SP-A sufficiency**			
Genes	Log2 fold change	Genes	Log2 fold change
HIF-1 signaling pathway		MAPK signaling pathway	
Egln1	-1.51	Met	-1.16
Ldha	-0.78	Map2k6	-2.15
Pgk1	-1.27	Dusp9	-1.78
Vegfa	-0.83	Arrb1	-1.48
Eno1b	-1.16	Vegfa	-0.83
Mtor	-0.89	Map3k13	-2.27
Eno1	-1.00	Hspb1	-0.97
Pfkl	-1.03	Ereg	-0.75
Hmox1	-0.61	Cacnb3	-0.64
Serpine1	-0.74	Ngf	-0.73
Aldoa	-0.73	Hspa1a	-0.48
		Hspa1b	-0.46
		Map4k4	-0.45

SP-A KO macrophages infected with IAV up-regulated 552 genes and down-regulated 257 genes compared to IAV infected WT macrophages (Fig. 5C). Pathway analysis did not show any significant pathways associated with antiviral defense, but instead showed significant association with pathways such as focal adhesion, cell adhesion molecules, and leukocyte transendothelial migration. Down-regulated genes were associated with the cytokine-cytokine receptor interaction pathway (Tables 4 and 5).

**Table 4:** Pathways of genes altered by SP-A deficiency (vs. Tollip/SP-A sufficiency) in mouse lung macrophages infected with IAV

Signaling Pathways	Gene Count	%	Up- or Down- Regulated	*P* value
ECM-receptor interaction	11	5.7	Up	2.16E-05
Focal adhesion	17	8.8	Up	5.94E-05
Cell adhesion molecules (CAMs)	11	5.7	Up	3.48E-03
Leukocyte transendothelial migration	7	3.6	Up	3.52E-02
Cytokine-cytokine receptor interaction	8	4.0	Down	2.39E-02

**Table 5:** List of genes in selected pathways altered by SP-A deficiency (vs. Tollip/SP-A sufficiency) in mouse lung macrophages infected with IAV

**Up-regulated genes by SP-A deficiency vs. Tollip/SP-A sufficiency**			
Genes	Log2 fold change	Genes	Log2 fold change
Focal adhesion		Cell adhesion molecules (CAMs)	
Itga8	2.79	Icam2	1.50
Cav1	1.20	Itga8	2.79
Mylk	1.48	Esam	1.84
Lama5	1.45	Cldn5	1.95
Col4a2	1.40	Pecam1	1.07
Col4a1	1.29	Cdh5	1.71
Itga1	1.13	Ptprm	1.85
Prkcg	1.64	Ctla4	0.93
Col6a2	1.28	Vcam1	0.72
Pdgfra	0.91	Lrrc4b	5.83
Vwf	0.96	Cadm1	1.18
Pdgfd	1.73		
Lamc3	1.69	Leukocyte transendothelial migration	
Col6a1	1.22	Esam	1.84
Vegfd	1.46	Cldn5	1.95
Col6a3	1.01	Pecam1	1.07
Mylk3	5.65	Rapgef3	1.51
		Cdh5	1.71
		Prkcg	1.64
		Vcam1	0.72
**Down-regulated genes by SP-A deficiency vs. Tollip/SP-A sufficiency**			
Cytokine-cytokine receptor interaction			
Genes	Log2 fold change	Genes	Log2 fold change
Ccr9	-2.29	Tnfrsf11b	-1.77
Cxcl5	-2.10	Il12rb2	-1.08
Tnfrsf9	-1.81	Il1a	-0.83
Il2rb	-0.94	Inhba	-0.87

After IAV infection, dKO macrophages up-regulated 241 genes and down-regulated 447 genes (Fig. 5D) compared to IAV infected WT macrophages. Cytokine-cytokine receptor interactions was one of the few significant pathways associated with the up-regulated genes. Importantly, the chemokine signaling pathway was significantly associated with the down-regulated genes (Tables 6 and 7).

**Table 6:** Pathways of genes altered by Tollip/SP-A deficiency (vs. Tollip/SP-A sufficiency) in mouse lung macrophages infected with IAV

**Signaling Pathways**	**Gene Count**	**%**	**Up- or Down- Regulated**	***P* value**
Cytokine-cytokine receptor interaction	6	0.3	Up	1.93E-02
Proteoglycans in cancer	5	0.3	Up	2.53E-02
African trypanosomiasis	2	0.1	Up	3.14E-02
Tyrosine metabolism	2	0.1	Up	3.32E-02
Graft-versus-host disease	3	0.1	Up	8.03E-03
Autoimmune thyroid disease	3	0.1	Up	1.22E-02
Chemokine signaling pathway	10	6.6	Down	8.64E-03
Cell adhesion molecules (CAMs)	8	5.3	Down	1.93E-02
ABC transporters	4	2.6	Down	1.75E-02
Citrate cycle (TCA cycle)	3	2.0	Down	3.50E-02
Biosynthesis of amino acids	11	7.2	Down	8.17E-07
Glycolysis / Gluconeogenesis	10	6.6	Down	1.38E-06
Carbon metabolism	10	6.6	Down	2.87E-04
Pyruvate metabolism	5	3.3	Down	1.38E-03
HIF-1 signaling pathway	8	5.3	Down	2.40E-03

**Table 7:** List of genes in selected pathways altered by Tollip/SP-A deficiency (vs. Tollip/SP-A sufficiency) in mouse lung macrophages infected with IAV

**Up-regulated genes by Tollip/SP-A deficiency vs. Tollip/SP-A sufficiency**			
Cytokine-cytokine receptor interaction			
Genes	Log2 fold change	Genes	Log2 fold change
Il18rap	0.99	Fasl	1.27
Ccl21a	1.91	Il5ra	1.96
Gm10591	1.47	Il18	1.58
**Down-regulated genes by Tollip/SP-A deficiency vs. Tollip/SP-A sufficiency**			
Chemokine signaling pathway			
Genes	Log2 fold change	Genes	Log2 fold change
Cxcl15	-1.88	Grk1	-6.56
Ccl24	-2.27	Ccl9	-1.07
Cxcl3	-1.52	Cxcl14	-1.44
Cxcl5	-1.95	Arrb1	-1.56
Ccl22	-1.31	Ccr9	-1.10

dKO (vs. Tollip KO) macrophages had 644 up-regulated genes, and 689 down-regulated genes (Fig. 5E). Influenza A and asthma pathways were significantly up-regulated in dKO macrophages as compared to Tollip KO macrophages (Tables 8 and 9). It is important to note, that of the down-regulated genes, those associated with the chemokine signaling pathway (CXCL5, Ccl9, Ccl12) and the IL-17 signaling pathway (IL-17) were significantly reduced in dKO macrophages compared to Tollip KO macrophages.

**Table 8:** Pathways of genes altered by Tollip/SP-A deficiency (vs. Tollip deficiency) in mouse lung macrophages infected with IAV

Signaling Pathways	Gene Count	%	Up- or Down- Regulated	*P* value
Notch signaling pathway	7	3.0	Up	4.31E-04
Longevity regulating pathway – multiple species	7	3.0	Up	1.80E-03
FoxO signaling pathway	11	4.7	Up	6.32E-03
Asthma	4	1.7	Up	7.45E-03
mTOR signaling pathway	12	5.1	Up	7.47E-03
TGF-beta signaling pathway	8	3.4	Up	7.49E-03
ECM-receptor interaction	8	3.4	Up	7.70E-03
Influenza A	12	5.1	Up	9.59E-03
Antigen processing and presentation	7	3.0	Up	1.56E-02
Wnt signaling pathway	10	4.2	Up	2.81E-02
Cell cycle	9	3.8	Up	3.11E-02
Oxidative phosphorylation	9	3.8	Up	4.26E-02
Cell adhesion molecules (CAMs)	21	7.7	Down	3.96E-07
Cytokine-cytokine receptor interaction	26	9.6	Down	1.69E-05
Hematopoietic cell lineage	12	4.4	Down	2.40E-04
Complement and coagulation cascades	10	3.7	Down	8.34E-04
IL-17 signaling pathway	11	4.1	Down	1.02E-03
T cell receptor signaling pathway	10	3.7	Down	8.07E-03
Chemokine signaling pathway	15	5.5	Down	8.11E-03
Primary immunodeficiency	5	1.8	Down	1.27E-02
ECM-receptor interaction	8	3.0	Down	2.06E-02
Adipocytokine signaling pathway	7	2.6	Down	2.44E-02
Th1 and Th2 cell differentiation	8	3.0	Down	2.67E-02
Nitrogen metabolism	3	1.1	Down	2.70E-02

**Table 9:** List of genes in selected pathways altered by Tollip/SP-A deficiency (vs. Tollip deficiency) in mouse lung macrophages infected with IAV

**Up-regulated genes by Tollip/SP-A deficiency vs. Tollip deficiency**			
Genes	Log2 fold change	Genes	Log2 fold change
Influenza A		Asthma	
Hspa1a	0.61	Fcer1g	0.76
Hspa1b	0.62	Il10	0.61
Hspa2	0.60	H2-Eb1	0.37
H2-Eb1	0.37	H2-DMb2	0.49
Ifna1	5.46		
Ivns1abp	0.29		
H2-DMb2	0.49		
Ep300	0.37		
Nup98	0.29		
Eif2ak4	0.45		
Nxf1	0.30		
Crebbp	0.32		
**Down-regulated genes by Tollip/SP-A deficiency vs. Tollip deficiency**			
Genes	Log2 fold change	Genes	Log2 fold change
Cytokine-cytokine receptor interaction		IL-17 signaling pathway	
Ccl9	-0.98	Lcn2	-0.93
Ccl12	-0.94	Ccl12	-0.94
Il1Rl1	-1.00	Csf2	-0.87
Ccr8	-1.28	Il17f	-3.31
Csf2	-0.87	Mapk10	-4.39
Il17f	-3.31	Ikbke	-0.50
Cxcl15	-2.00	Mapk8	-0.54
Gdf3	-1.59	Cxcl5	-0.61
Ccl1	-1.40	Ccl2	-0.34
Inhba	-0.46	Mmp13	-0.78
Inhbb	-1.13	Il5	-1.03
Il1a	-0.33		
Il34	-2.59	Chemokine signaling pathway	
Cxcl9	-0.46	Ccl9	-0.98
Cx3cl1	-0.59	Ccl12	-0.94
Cxcr6	-0.61	Ccr8	-1.28
Cxcl5	-0.61	Cxcl15	-2.00
Ccr4	-0.67	Ccl1	-1.40
Il22ra1	-5.20	Cxcl9	-0.46
Ccl2	-0.34	Cx3cl1	-0.59
Il5	-1.03	Cxcr6	-0.61
Ccr5	-0.32	Cxcl5	-0.61
Ccr1	-0.34	Ccr4	-0.67
Bmp6	-0.61	Ccl2	-0.34
Ccl5	-0.32	Ccr5	-0.32
Acvrl1	-0.27	Ccr1	-0.34
		Ccl5	-0.32
		Gnb4	-0.51

dKO (vs. SP-A KO) macrophages had 698 up-regulated genes, and 1050 down-regulated genes (Fig. 5F). Influenza A and cytokine-cytokine receptor interaction pathways were significantly higher in dKO macrophages as compared to SP-A KO macrophages (Supplementary Tables 3 and 4). Of the down-regulated genes, those associated with focal adhesion and cell adhesion molecules were significantly reduced in dKO macrophages compared to SP-A KO macrophages.

These data suggest SP-A deficiency in the absence of Tollip further inhibits the appropriate antiviral inflammatory response involved in neutrophil chemokine signaling.

## Discussion

For the first time we have shown that the deficiency of both Tollip and SP-A during an IAV infection contributed to increased viral load associated with lack of robust lung immune response such as neutrophil recruitment. We have also shown that SP-A may have therapeutic implication in restoring antiviral function in lung tissues with both Tollip and SP-A deficiency.

Our group and others have shown that Tollip regulates virus inflammation by directly interacting with other proteins such as STING, or by indirectly enhancing anti-inflammatory protein expression [7, 8, 10, 14]. However, whether there is cooperation of Tollip and SP-A in vivo during IAV infection remained unclear. Our preliminary experiment (data not shown here) suggests that these two modulators may maintain each other’s levels after exposure to various stimuli. Briefly, Tollip KO mice infected with rhinovirus had less SP-A in BAL fluid compared to WT mice, while IL-13-treated SP-A KO mice had significantly less Tollip expression than WT mice. This report demonstrates that sufficient levels of both Tollip and SP-A are critical to reduce the severity of IAV infection in part through maintaining a proper inflammatory response. This novel finding was supported by our observation that dKO mice increased viral load in the lung tissue and BAL fluid, which is uncoupled with IFN-beta mRNA expression and lung neutrophil recruitment. Appropriate levels of neutrophils are vital to mount an antiviral inflammatory response, and type I interferon signaling is critical to the maturation and survival of neutrophils [55]. Neutrophils contribute to viral clearance through engulfment of viruses, release of antimicrobial substances and neutrophil extracellular traps (NETs) [56]. Previous clinical studies found that 10 to 18% of patients with influenza virus infection developed transient neutropenia [57] and that patients with neutropenia had high rates of influenza complications [58]. These clinical findings further support the role of appropriate levels of neutrophils in host defense against IAV infection. However, excessive neutrophil recruitment may lead to tissue injury from neutrophil elastase and oxidative damage [50]. Together, while SP-A KO mice with sufficient Tollip expression did not demonstrate increased risk of IAV infection, SP-A deficiency in hosts with lower levels of Tollip may be at a higher risk of more severe IAV infection in part due to reduced neutrophil recruitment or activation. It is important to note, we found that viral load differences between dKO and Tollip KO mice was greater in BAL fluid than in the lung tissue. While the exact mechanisms remain unclear, we propose that SP-A/Tollip deficiency may primarily impair the clearance of viruses that were released from lung tissue cells and subsequently increase the transmission or spread of the viruses to neighboring tissues or cells. It appeared that SP-A/Tollip deficiency has a minimal role in modulating tissue viral load or viral replication.

One of the important questions unsolved in this study is why SP-A deficiency alone did not impair innate immunity. Our findings showed SP-A KO mice had similar lung tissue IAV RNA levels to the dKO mice. However, the plaque assay data showed that SP-A KO mice had significantly less IAV PFU/ml than dKO mice. This suggests that the infectivity of IAV from SP-A KO mice is less than that from dKO mice. How SP-A affects viral infection has not been well understood. Previous work regarding the role of SP-A during viral infection has focused primarily on its ability to bind to virus resulting in either increased phagocytosis or neutralization of the virus particle. One study found that incubation of macrophages with SP-A before, during, or after IAV infection suppressed the infection [54]. Interestingly, SP-A deficiency coupled with Tollip deficiency (vs. Tollip deficiency alone) demonstrated lack of a robust immune response to IAV with increased viral load. When IFN-beta mRNA expression was measured, Tollip KO mice infected with IAV had significantly higher levels, which may serve as a mechanism to reduce the severity of viral infection in the presence of SP-A. However, dKO mice with the highest level of viruses in BAL fluid were unable to increase IFN-beta levels, which may serve as an additional mechanism for increased viral load. We also identified lack of robust induction of neutrophil chemokine LIX in BAL fluid of dKO mice. Furthermore, our RNA-seq data in lung macrophages supported that dKO mice demonstrated decreased expression of genes associated with neutrophil recruitment and activation (i.e., CXCL5, IL-17) compared to WT and Tollip KO mice. This data suggests that while dKO mice have an enhanced response to viral infection, they are not able to elicit a robust neutrophilic response. Since IAV-infected dKO mice showed the phenotype of less neutrophil inflammation, less LIX release, and more viral burden, administration of recombinant LIX may be beneficial to reverse the phenotype. The exact molecular mechanisms by which SP-A and Tollip cooperate in IAV infection remains to be explored in future studies. One such study could determine if both SP-A and Tollip are necessary in modulating endosomal trafficking and function [54, 59], which is important in viral entry and recognition by the cell [60]. Another potential mechanism is that the combination of Tollip and SP-A stabilize other immune proteins such as STING [61], which has been shown to activate an antiviral response following mitochondrial DNA release caused by IAV infection [62].

Having shown the dependency of SP-A function on Tollip during IAV infection, we explored if SP-A may have the therapeutic implication in restoring antiviral function in lungs with both SP-A and Tollip deficiency. By leveraging the PCLS model where interactions of various cell types are maintained, we tested if exogenous SP-A treatment could inhibit IAV infection. One of the interesting findings in our study is that while the PCLS tissue viral data (Fig. 3A) in dKO vs. WT mice was similar to the in vivo data, the dKO PCLS supernatant data showed a slight decrease in viral release compared to WT slices, whereas in vivo, dKO mice had more viral load released into their BAL fluid compared to WT mice. One explanation for such differences could be the lack of mucociliary mechanisms in PCLS. In vivo*,* mucociliary mechanism exists, which may be compromised in dKO mice. In PCLS, dKO PCLS increased IFN-β likely due to increased lung tissue viral load. The increased IFN-β may in turn reduce the release of intracellular viruses into the extracellular space. Additionally, recombinant SP-A decreased viral load in PCLS of dKO mice. Our data was in line with a study conducted by Al-Qahtani et al. who showed that full-length SP-A added to A594 cells infected with IAV decreased viral load as well as pro-inflammatory cytokines [19]. To determine the mechanism of how recombinant SP-A inhibits viral infection, we measured viral load in slices treated with or without SP-A over a period of time. Our data suggests that SP-A did not inhibit viral entry into the cell, but rather decreased viral replication once inside the cell. How SP-A inhibits viral replication in lung tissue remains to be determined. A study by Yau et al. reported that SP-A inhibited macrophage IAV infection by delaying IAV trafficking to the endosomes where viral hemagglutinin (HA) undergoes conformational necessary to allow viral proteins to enter the cytosol and transport to the nucleus for viral replication [54]. We then measured TNF mRNA expression in PCLS as TNF is a pro-inflammatory cytokine produced primarily by macrophages [63–65], and macrophages represent a major cell type in PCLS tissue [66]. Interestingly, SP-A increased TNF-a expression in dKO PCLS. Since macrophages was previously shown to enhance IAV clearance, our findings further support the notion that SP-A administration may be beneficial to reduce the severity of IAV infection in hosts with both SP-A and Tollip deficiency. It is important to note that SP-A treatment did not rescue LIX levels in dKO slices. While LIX is produced by a variety of cells including epithelial cells, keratinocytes, endothelial cells, fibroblasts, neutrophils, and monocytes [67] the lack of migrating neutrophils in our PCLS model could explain the similar levels of LIX seen in IAV-infected lung slices with and without SP-A treatment. Furthermore, we did not restore Tollip expression in dKO PCLS, which suggests the cooperative effect of both SP-A and Tollip in antiviral inflammatory responses.

There are several limitations to this study. First, our IAV model does not include allergen challenges to determine the role of Tollip/SP-A deficiency in asthma exacerbations. Second, this study only focused on a very acute IAV infection. It remains unclear if Tollip/SP-A deficiency prolongs IAV infection or delays clearance at a later time, therefore additional time points should be considered. Third, our data shows that the reduction in viral load seen in SP-A treated PCLS is likely due to SP-A-mediated inhibition of viral replication, however, the underlying mechanism needs to be further investigated. Fourth, whether the decrease in genes associated with chemokine signaling seen in dKO macrophages leads to impaired neutrophil recruitment and viral clearance could be explored. Fifth, the exact mechanism of how Tollip/SP-A double deficiency inhibited chemokine transcription and the ensuing neutrophil recruitment has yet to be elucidated. Lastly, how these two immune modulators work together, whether through direct interaction or indirectly, to reduce inflammation needs to be further determined.

## Conclusion

**Fig. 6:**
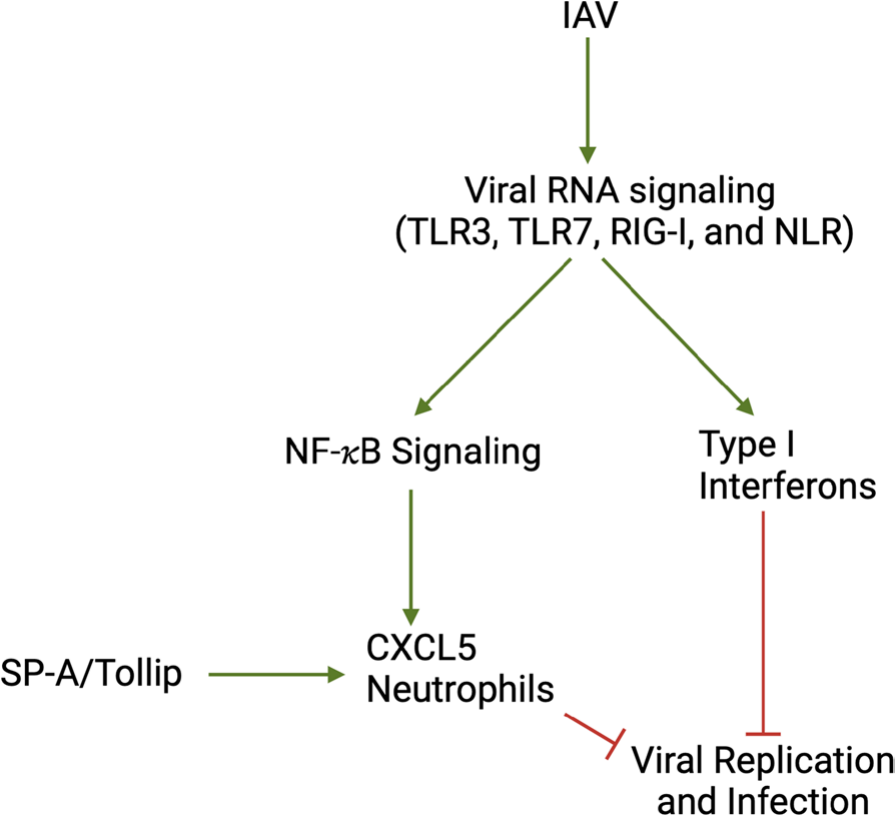
Proposed mechanisms underlying Tollip and SP-A cooperation to modulate IAV infection and pro-inflammatory response. In Tollip- and SP-A-sufficient lung (e.g., epithelial cells and macrophages), IAV infection increases interferon (e.g., type I interferon) expression and chemokines via viral RNA signaling to effectively clear the viruses. In the absence of both Tollip and SP-A, the lung fails to clear the viruses partly due to impaired generation of antiviral mediators (e.g., type I interferons and chemokines) and lack appropriate levels of neutrophil recruitment. RIG-I = retinoic acid-inducible gene I. NLR = NOD like receptor

By utilizing our novel dKO mouse strain for both in vivo IAV infections as well as PCLS cultures, we have demonstrated that Tollip/SP-A deficiency enhances IAV infection via an impaired innate immune response (i.e., neutrophilic inflammation, Fig. 6), and that SP-A’s role in viral infection is dependent on the presence of Tollip. Supplementation of SP-A or enhancement of Tollip expression or function in hosts with SP-A deficiency may provide a new approach to attenuate the severity of IAV infection.

### Supplementary Information

**Additional file 1.** WT IAV vs WT PBS – downregulated genes. A table of genes downregulated by IAV in WT mouse macrophages, with the log2 fold change, and *p*-values.

**Additional file 2.** WT IAV vs WT PBS – upregulated genes. A table of genes upregulated by IAV in WT mouse macrophages, with the log2 fold change, and *p*-values.

**Additional file 3.** SP-A KO IAV vs WT IAV – downregulated genes. A table of genes that are downregulated in IAV infected SP-A KO mouse macrophages compared to IAV infected WT macrophages. This includes the log2 fold change and *p*-values.

**Additional file 4.** SP-A KO IAV vs WT IAV – upregulated genes. A table of genes that are upregulated in IAV infected SP-A KO macrophages compared to IAV infected WT macrophages. This includes the log2 fold change and *p*-values.

**Additional file 5.** Tollip KO IAV vs WT IAV – downregulated genes. A table of genes that are downregulated in IAV infected Tollip KO macrophages compared to IAV infected WT macrophages. This includes the log2 fold change and *p*-values.

**Additional file 6.** Tollip KO IAV vs WT IAV – upregulated genes. A table of genes that are upregulated in IAV infected Tollip KO macrophages compared to IAV infected WT macrophages. This includes the log2 fold change and *p*-values.

**Additional file 7.** dKO IAV vs WT IAV – downregulated genes. A table of genes that are downregulated in IAV infected dKO macrophages compared to IAV infected WT macrophages. This includes the log2 fold change and *p*-values.

**Additional file 8.** dKO IAV vs WT IAV – upregulated genes. A table of genes that are upregulated in IAV infected dKO macrophages compared to IAV infected WT macrophages. This includes the log2 fold change and *p*-values.

**Additional file 9.** dKO IAV vs Tollip KO IAV – downregulated genes. A table of genes that are downregulated in IAV infected dKO macrophages compared to IAV infected Tollip KO macrophages. This includes the log2 fold change and *p*-values.

**Additional file 10.** dKO IAV vs Tollip KO IAV – upregulated genes. A table of genes that are upregulated in IAV infected dKO macrophages compared to IAV infected Tollip KO macrophages. This includes the log2 fold change and *p*-values.

**Additional file 11: Supplementary Figure 1.** Tollip/SP-A genotyping confirmation. (A) Tollip sufficient mice have a single band around 600 base pairs, and Tollip deficient mice have a single band around 1,100 bp. (B) SP-A sufficient mice have a single band around 167 bp, ad SP-A deficient mice have a single band around 320 bp.

**Additional file 12: Supplementary Table 1.** Pathways of genes altered by IAV infection (vs. PBS control) in wild-type (Tollip/SP-A sufficient) mouse lung macrophages. A table of signaling pathways, the gene count, the percent up- or down- regulated, and the *p*-value of important pathways found to be associated in mouse lung macrophages.

**Additional file 13: Supplementary Table 2.** List of genes in selected pathways altered by IAV infection (vs. PBS control) in wild-type (Tollip/SP-A sufficient) mouse lung macrophages. A table of genes and the log2 fold change associated with the pathways listed in Supplementary table 1.

**Additional file 14: Supplementary Table 3.** Pathways of genes altered by Tollip/SP-A deficiency (vs. SP-A deficiency) in mouse lung macrophages infected with IAV. A table of signaling pathways, the gene count, the percent up- or down- regulated, and the *p*-value of important pathways found to be associated in mouse lung macrophages.

**Additional file 15: Supplementary Table 4.** List of genes in selected pathways altered by Tollip/SP-A deficiency (vs. SP-A deficiency) in mouse lung macrophages infected with IAV. A table of genes and the log2 fold change associated with the pathways listed in Supplementary table 3.

## Data Availability

No datasets were generated or analysed during the current study.

## Abbreviations:

IAV: Influenza A Virus
Tollip: Toll-interacting protein
SP-A: Surfactant Protein A
CRD: Carbohydrate recognition domain
KO: Knockout
WT: Wild-type
dKO: Double knockout
PCLS: Precision cut lung slices

## References

[1] Shrestha SS, Swerdlow DL, Borse RH, Prabhu VS, Finelli L, Atkins CY (2011). Estimating the burden of 2009 pandemic influenza A (H1N1) in the United States (April 2009-April 2010). Clin Infect Dis.

[2] Veerapandian R, Snyder JD, Samarasinghe AE (2018). Influenza in asthmatics: for better or for worse?. Front Immunol.

[3] LeMessurier KS, Rooney R, Ghoneim HE, Liu B, Li K, Smallwood HS (2020). Influenza A virus directly modulates mouse eosinophil responses. J Leukoc Biol.

[4] Liu B, Li NL, Shen Y, Bao X, Fabrizio T, Elbahesh H (2016). The C-terminal tail of TRIM56 dictates antiviral restriction of influenza A and B viruses by impeding viral RNA synthesis. J Virol.

[5] Li X, Goobie GC, Gregory AD, Kass DJ, Zhang Y (2021). Toll-interacting protein in pulmonary diseases. Abiding by the goldilocks principle. Am J Respir Cell Mol Biol.

[6] Colotta F, Lampugnani MG, Polentarutti N, Dejana E, Mantovani A (1988). Interleukin-1 induces c-fos protooncogene expression in cultured human endothelial cells. Biochem Biophys Res Commun.

[7] Schaunaman N, Dimasuay KG, Cervantes D, Li L, Numata M, Kraft M (2023). Tollip inhibits IL-33 release and inflammation in influenza A virus-infected mouse airways. J Innate Immun..

[8] Schaunaman N, Dimasuay KG, Kraft M, Chu HW (2022). Tollip interaction with STAT3: a novel mechanism to regulate human airway epithelial responses to type 2 cytokines. Respir Res.

[9] Ito Y, Schaefer N, Sanchez A, Francisco D, Alam R, Martin RJ (2018). Toll-Interacting protein, Tollip, inhibits IL-13-mediated pulmonary eosinophilic inflammation in mice. J Innate Immun.

[10] Dakhama A, Al Mubarak R, Pavelka N, Voelker D, Seibold M, Ledford JG (2020). Tollip inhibits ST2 signaling in airway epithelial cells exposed to type 2 cytokines and rhinovirus. J Innate Immun.

[11] Huang C, Jiang D, Francisco D, Berman R, Wu Q, Ledford JG (2016). Tollip SNP rs5743899 modulates human airway epithelial responses to rhinovirus infection. Clin Exp Allergy.

[12] Szklarczyk D, Gable AL, Nastou KC, Lyon D, Kirsch R, Pyysalo S (2021). The STRING database in 2021: customizable protein-protein networks, and functional characterization of user-uploaded gene/measurement sets. Nucleic Acids Res.

[13] Didierlaurent A, Brissoni B, Velin D, Aebi N, Tardivel A, Kaslin E (2006). Tollip regulates proinflammatory responses to interleukin-1 and lipopolysaccharide. Mol Cell Biol.

[14] Zhang G, Ghosh S (2002). Negative regulation of toll-like receptor-mediated signaling by Tollip. J Biol Chem.

[15] Burns K, Clatworthy J, Martin L, Martinon F, Plumpton C, Maschera B (2000). Tollip, a new component of the IL-1RI pathway, links IRAK to the IL-1 receptor. Nat Cell Biol.

[16] Nayak A, Dodagatta-Marri E, Tsolaki AG, Kishore U (2012). An insight into the diverse roles of surfactant proteins, SP-A and SP-D in innate and adaptive immunity. Front Immunol.

[17] Khubchandani KR, Snyder JM (2001). Surfactant protein A (SP-A): the alveolus and beyond. FASEB J.

[18] Benne CA, Kraaijeveld CA, van Strijp JA, Brouwer E, Harmsen M, Verhoef J (1995). Interactions of surfactant protein A with influenza A viruses: binding and neutralization. J Infect Dis.

[19] Al-Qahtani AA, Murugaiah V, Bashir HA, Pathan AA, Abozaid SM, Makarov E (2019). Full-length human surfactant protein A inhibits influenza A virus infection of A549 lung epithelial cells: A recombinant form containing neck and lectin domains promotes infectivity. Immunobiology.

[20] Wang Y, Voelker DR, Lugogo NL, Wang G, Floros J, Ingram JL (2011). Surfactant protein A is defective in abrogating inflammation in asthma. Am J Physiol Lung Cell Mol Physiol.

[21] Benne CA, Benaissa-Trouw B, van Strijp JA, Kraaijeveld CA, van Iwaarden JF (1997). Surfactant protein A, but not surfactant protein D, is an opsonin for influenza A virus phagocytosis by rat alveolar macrophages. Eur J Immunol.

[22] Lugogo N, Francisco D, Addison KJ, Manne A, Pederson W, Ingram JL (2018). Obese asthmatic patients have decreased surfactant protein A levels: mechanisms and implications. J Allergy Clin Immunol..

[23] Herrera-Ramos E, Lopez-Rodriguez M, Ruiz-Hernandez JJ, Horcajada JP, Borderias L, Lerma E (2014). Surfactant protein A genetic variants associate with severe respiratory insufficiency in pandemic influenza A virus infection. Crit Care.

[24] LeVine AM, Hartshorn K, Elliott J, Whitsett J, Korfhagen T (2002). Absence of SP-A modulates innate and adaptive defense responses to pulmonary influenza infection. Am J Physiol Lung Cell Mol Physiol.

[25] Li G, Siddiqui J, Hendry M, Akiyama J, Edmondson J, Brown C (2002). Surfactant protein-A–deficient mice display an exaggerated early inflammatory response to a beta-resistant strain of influenza A virus. Am J Respir Cell Mol Biol.

[26] Chroneos ZC, Sever-Chroneos Z, Shepherd VL (2010). Pulmonary surfactant: an immunological perspective. Cell Physiol Biochem.

[27] Daly K, Nguyen P, Woodland DL, Blackman MA (1995). Immunodominance of major histocompatibility complex class I-restricted influenza virus epitopes can be influenced by the T-cell receptor repertoire. J Virol.

[28] Hartshorn KL, White MR, Tecle T, Holmskov U, Crouch EC (2006). Innate defense against influenza A virus: activity of human neutrophil defensins and interactions of defensins with surfactant protein D. J Immunol.

[29] Numata M, Mitchell JR, Tipper JL, Brand JD, Trombley JE, Nagashima Y (2020). Pulmonary surfactant lipids inhibit infections with the pandemic H1N1 influenza virus in several animal models. J Biol Chem.

[30] Kunisaki KM, Janoff EN (2009). Influenza in immunosuppressed populations: a review of infection frequency, morbidity, mortality, and vaccine responses. Lancet Infect Dis.

[31] Chan RW, Yuen KM, Yu WC, Ho CC, Nicholls JM, Peiris JS (2010). Influenza H5N1 and H1N1 virus replication and innate immune responses in bronchial epithelial cells are influenced by the state of differentiation. PLoS ONE.

[32] Chen K, Yuan R, Zhang Y, Geng S, Li L (2017). Tollip deficiency alters atherosclerosis and steatosis by disrupting lipophagy. J Am Heart Assoc.

[33] Francisco D, Wang Y, Conway M, Hurbon AN, Dy ABC, Addison KJ (2020). Surfactant Protein-A protects against IL-13-Induced inflammation in asthma. J Immunol.

[34] Korfhagen TR, Bruno MD, Ross GF, Huelsman KM, Ikegami M, Jobe AH (1996). Altered surfactant function and structure in SP-A gene targeted mice. Proc Natl Acad Sci U S A.

[35] Nouri HR, Schaunaman N, Kraft M, Li L, Numata M, Chu HW (2023). Tollip deficiency exaggerates airway type 2 inflammation in mice exposed to allergen and influenza A virus: role of the ATP/IL-33 signaling axis. Front Immunol.

[36] Dimasuay KG, Berg B, Schaunaman N, Chu HW (2023). Role of myeloid cell-specific TLR9 in mitochondrial DNA-induced lung inflammation in mice. Int J Mol Sci..

[37] Somerville L, Cardani A, Braciale TJ (2020). Alveolar macrophages in influenza A infection guarding the castle with sleeping dragons. Infect Dis Ther (San Antonio)..

[38] Jacob IB, Gemmiti A, Xiong W, Reynolds E, Nicholas B, Thangamani S (2024). Human surfactant protein A alleviates SARS-CoV-2 infectivity in human lung epithelial cells. Front Immunol..

[39] Agraval H, Crue T, Schaunaman N, Numata M, Day BJ, Chu HW (2023). Electronic cigarette exposure increases the severity of influenza a virus infection via TRAIL dysregulation in human precision-cut lung slices. Int J Mol Sci.

[40] Chen Y, Cui D, Zheng S, Yang S, Tong J, Yang D (2011). Simultaneous detection of influenza A, influenza B, and respiratory syncytial viruses and subtyping of influenza A H3N2 virus and H1N1 (2009) virus by multiplex real-time PCR. J Clin Microbiol.

[41] da Huang W, Sherman BT, Lempicki RA (2009). Systematic and integrative analysis of large gene lists using DAVID bioinformatics resources. Nat Protoc.

[42] da Huang W, Sherman BT, Lempicki RA (2009). Bioinformatics enrichment tools: paths toward the comprehensive functional analysis of large gene lists. Nucleic Acids Res.

[43] Todd EM, Zhou JY, Szasz TP, Deady LE, D'Angelo JA, Cheung MD (2016). Alveolar macrophage development in mice requires L-plastin for cellular localization in alveoli. Blood.

[44] Hu G, Christman JW (2019). Editorial: alveolar macrophages in lung inflammation and resolution. Front Immunol.

[45] Dewhurst JA, Lea S, Hardaker E, Dungwa JV, Ravi AK, Singh D (2017). Characterisation of lung macrophage subpopulations in COPD patients and controls. Sci Rep.

[46] Berkebile AR, Bartlett JA, Abou Alaiwa M, Varga SM, Power UF, McCray PB (2020). Airway surface liquid has innate antiviral activity that is reduced in cystic fibrosis. Am J Respir Cell Mol Biol.

[47] Campione E, Lanna C, Cosio T, Rosa L, Conte MP, Iacovelli F (2021). Lactoferrin as antiviral treatment in COVID-19 management: preliminary evidence. Int J Environ Res Public Health.

[48] Malaczewska J, Kaczorek-Lukowska E, Wojcik R, Siwicki AK (2019). Antiviral effects of nisin, lysozyme, lactoferrin and their mixtures against bovine viral diarrhoea virus. BMC Vet Res.

[49] Hashimoto Y, Moki T, Takizawa T, Shiratsuchi A, Nakanishi Y (2007). Evidence for phagocytosis of influenza virus-infected, apoptotic cells by neutrophils and macrophages in mice. J Immunol.

[50] Ma Y, Zhang Y, Zhu L (2021). Role of neutrophils in acute viral infection. Immun Inflamm Dis.

[51] Shibayama N, Imai K, Morimoto H, Saigo S (1995). Oxygen equilibrium properties of nickel(II)-iron(II) hybrid hemoglobins cross-linked between 82 beta 1 and 82 beta 2 lysyl residues by bis(3,5-dibromosalicyl)fumarate: determination of the first two-step microscopic Adair constants for human hemoglobin. Biochemistry.

[52] Mattner J, Schindler H, Diefenbach A, Rollinghoff M, Gresser I, Bogdan C (2000). Regulation of type 2 nitric oxide synthase by type 1 interferons in macrophages infected with Leishmania major. Eur J Immunol.

[53] Perrone LA, Belser JA, Wadford DA, Katz JM, Tumpey TM (2013). Inducible nitric oxide contributes to viral pathogenesis following highly pathogenic influenza virus infection in mice. J Infect Dis.

[54] Yau E, Yang L, Chen Y, Umstead TM, Atkins H, Katz ZE (2023). Surfactant protein A alters endosomal trafficking of influenza A virus in macrophages. Front Immunol.

[55] Siakaeva E, Pylaeva E, Spyra I, Bordbari S, Hoing B, Kurten C (2019). Neutrophil maturation and survival is controlled by IFN-dependent regulation of NAMPT signaling. Int J Mol Sci.

[56] Schultz BM, Acevedo OA, Kalergis AM, Bueno SM (2022). Role of extracellular trap release during bacterial and viral infection. Front Microbiol.

[57] Higgins P, Runnegar N, Bird RJ, Markey KA (2016). Rates of neutropenia in adults with influenza A or B: a retrospective analysis of hospitalised patients in South East Queensland during 2015. Intern Med J.

[58] Durani U, Dioverti Prono MV, Tosh PK, Patnaik M, Barreto JN, Tande AJ (2017). Influenza infection in neutropenic adults. Infect Dis (Lond).

[59] Matsushita H, Kurihara N, Wakayama K, Fujimoto S, Kanazawa H, Fujiwara H (1992). Dyspnea and ventilatory muscle function during exercise on air and oxygen breathing in patients with chronic obstructive pulmonary disease (COPD). Nihon Kyobu Shikkan Gakkai Zasshi.

[60] Mercer J, Schelhaas M, Helenius A (2010). Virus entry by endocytosis. Annu Rev Biochem.

[61] Pokatayev V, Yang K, Tu X, Dobbs N, Wu J, Kalb RG (2020). Homeostatic regulation of STING protein at the resting state by stabilizer TOLLIP. Nat Immunol.

[62] Moriyama M, Koshiba T, Ichinohe T (2019). Influenza A virus M2 protein triggers mitochondrial DNA-mediated antiviral immune responses. Nat Commun.

[63] Wajant H, Pfizenmaier K, Scheurich P (2003). Tumor necrosis factor signaling. Cell Death Differ.

[64] Flynn JL, Goldstein MM, Chan J, Triebold KJ, Pfeffer K, Lowenstein CJ (1995). Tumor necrosis factor-alpha is required in the protective immune response against Mycobacterium tuberculosis in mice. Immunity.

[65] Pfeffer K, Matsuyama T, Kundig TM, Wakeham A, Kishihara K, Shahinian A (1993). Mice deficient for the 55 kd tumor necrosis factor receptor are resistant to endotoxic shock, yet succumb to L. monocytogenes infection. Cell.

[66] Lyons-Cohen MR, Thomas SY, Cook DN, Nakano H (2017). Precision-cut mouse lung slices to visualize live pulmonary dendritic cells. J Vis Exp.

[67] Deng J, Jiang R, Meng E, Wu H (2022). CXCL5: a coachman to drive cancer progression. Front Oncol.

