# Supplementary figures and images for: Cooperation of immune regulators Tollip and surfactant protein A inhibits influenza A virus infection in mice
Source: Respir Res. 2024 May 3;25:193. doi: 10.1186/s12931-024-02820-3 (PMC11068576; doi:10.1186/s12931-024-02820-3)

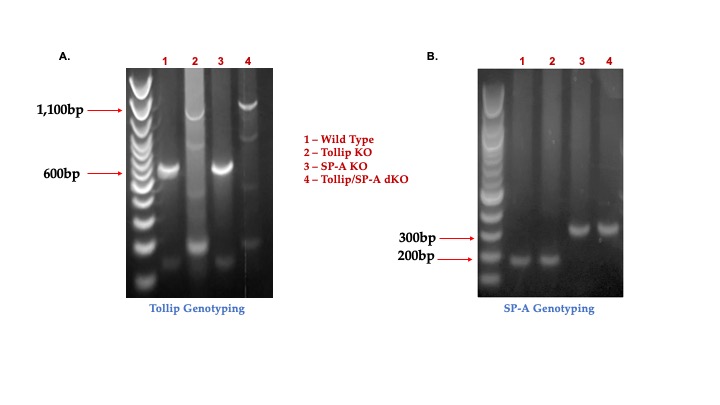

Supplement: Supplementary file 11 — Additional file 11: Supplementary Figure 1. Tollip/SP-A genotyping confirmation. (A) Tollip sufficient mice have a single band around 600 base pairs, and Tollip deficient mice have a single band around 1,100 bp. (B) SP-A sufficient mice have a single band around 167 bp, ad SP-A deficient mice have a single band around 320 bp. [file 12931_2024_2820_MOESM11_ESM.jpeg]
