# Supplementary material for: Cooperation of immune regulators Tollip and surfactant protein A inhibits influenza A virus infection in mice
Source: Respir Res. 2024 May 3;25:193. doi: 10.1186/s12931-024-02820-3 (PMC11068576; doi:10.1186/s12931-024-02820-3)
Supplement: Supplementary file 12 — Additional file 12: Supplementary Table 1. Pathways of genes altered by IAV infection (vs. PBS control) in wild-type (Tollip/SP-A sufficient) mouse lung macrophages. A table of signaling pathways, the gene count, the percent up- or down- regulated, and the p-value of important pathways found to be associated in mouse lung macrophages. [file 12931_2024_2820_MOESM12_ESM.docx]

**Supplementary Table 1.** Pathways of genes altered by IAV infection (vs. PBS control) in wild-type (Tollip/SP-A sufficient) mouse lung macrophages

| **Signaling Pathways** | **Gene Count** | **%** | **Up- or Down- Regulated** | **P value** |
| --- | --- | --- | --- | --- |
| Proteasome | 30 | 4.1 | Up | 2.46E-18 |
| NOD-like receptor signaling pathway | 44 | 6.1 | Up | 5.58E-09 |
| Antigen processing and presentation | 27 | 3.7 | Up | 7.91E-09 |
| Cytokine-cytokine receptor interaction | 60 | 8.3 | Up | 9.69E-09 |
| Phagosome | 40 | 5.5 | Up | 3.90E-07 |
| IL-17 signaling pathway | 26 | 3.6 | Up | 1.09E-06 |
| TNF signaling pathway | 30 | 4.1 | Up | 1.88E-06 |
| JAK-STAT signaling pathway | 36 | 5.0 | Up | 6.75E-06 |
| Influenza A | 36 | 5.0 | Up | 1.46E-05 |
| Apoptosis | 31 | 4.3 | Up | 3.50E-05 |
| Cell adhesion molecules (CAMs) | 33 | 4.5 | Up | 3.90E-05 |
| Toll-like receptor signaling pathway | 24 | 3.3 | Up | 5.39E-05 |
| RIG-I-like receptor signaling pathway | 17 | 2.3 | Up | 1.94E-04 |
| Natural killer cell mediated cytotoxicity | 25 | 3.4 | Up | 2.15E-04 |
| Cytosolic DNA-sensing pathway | 15 | 2.1 | Up | 7.13E-04 |
| Glycolysis / Gluconeogenesis | 16 | 2.2 | Up | 1.12E-03 |
| Chemokine signaling pathway | 34 | 4.7 | Up | 1.38E-03 |
| NF-kappa B signaling pathway | 19 | 2.6 | Up | 6.76E-03 |
|  |  |  |  |  |
| ECM-receptor interaction | 26 | 4.2 | Down | 1.43E-08 |
| Focal adhesion | 42 | 6.7 | Down | 3.12E-07 |
| Rap1 signaling pathway | 40 | 6.4 | Down | 2.94E-06 |
| Axon guidance | 36 | 5.8 | Down | 3.37E-06 |
| Gap junction | 19 | 3.0 | Down | 1.74E-04 |
| cGMP-PKG signaling pathway | 29 | 4.6 | Down | 3.83E-04 |
| Protein digestion and absorption | 18 | 2.9 | Down | 6.11E-04 |
| Tight junction | 27 | 4.3 | Down | 1.52E-03 |
| Hematopoietic cell lineage | 18 | 2.9 | Down | 1.67E-03 |
| Calcium signaling pathway | 28 | 4.5 | Down | 2.70E-03 |
| PI3K-Akt signaling pathway | 48 | 7.7 | Down | 3.05E-03 |
| cAMP signaling pathway | 29 | 4.6 | Down | 3.50E-03 |
