# Supplementary material for: Cooperation of immune regulators Tollip and surfactant protein A inhibits influenza A virus infection in mice
Source: Respir Res. 2024 May 3;25:193. doi: 10.1186/s12931-024-02820-3 (PMC11068576; doi:10.1186/s12931-024-02820-3)
Supplement: Supplementary file 13 — Additional file 13: Supplementary Table 2. List of genes in selected pathways altered by IAV infection (vs. PBS control) in wild-type (Tollip/SP-A sufficient) mouse lung macrophages. A table of genes and the log2 fold change associated with the pathways listed in Supplementary table 1. [file 12931_2024_2820_MOESM13_ESM.docx]

**Supplementary Table 2.** List of genes in selected pathways altered by IAV infection (vs. PBS control) in Tollip/SP-A sufficient mouse lung macrophages

| **Up-regulated genes by IAV vs. PBS control** | | | |
| --- | --- | --- | --- |
| Genes | Log2 fold change | Genes | Log2 fold change |
| IL-17 signaling pathway | | TNF signaling | |
| Cxcl5 | 5.55 | Ifi47 | 2.70 |
| Csf3 | 3.70 | Mlkl | 2.83 |
| Lcn2 | 2.43 | Gm5431 | 3.84 |
| Ccl2 | 2.02 | Mmp14 | 1.88 |
| Csf2 | 2.84 | Ccl2 | 2.02 |
| Cxcl3 | 2.24 | Csf2 | 2.84 |
| Ptgs2 | 1.63 | Cxcl3 | 2.24 |
| Ccl7 | 1.57 | Traf1 | 1.51 |
| Cxcl10 | 2.38 | Ptgs2 | 1.63 |
| Usp25 | 0.90 | Lif | 1.62 |
| Ikbke | 1.99 | Map2k1 | 1.22 |
| Mmp3 | 1.53 | Cxcl10 | 2.38 |
| Il1b | 1.85 | Mmp3 | 1.52 |
| Tradd | 1.03 | Rps6ka4 | 0.83 |
| Mmp13 | 4.18 | Il1b | 1.85 |
| Traf2 | 0.83 | Vcam1 | 0.86 |
| Casp8 | 0.76 | Tradd | 1.03 |
| Ifng | 2.53 | Irf1 | 1.25 |
| Fosl1 | 0.84 | Csf1 | 1.40 |
| Mapk7 | 0.68 | Ccl5 | 1.27 |
| Il6 | 0.81 | Traf2 | 0.83 |
| Casp3 | 0.68 | Casp8 | 0.76 |
| Traf3 | 0.64 | Map2k4 | 0.62 |
| Ccl12 | 4.19 | Il6 | 0.81 |
| Il5 | 1.17 | Ripk3 | 0.77 |
| Rela | 0.61 | Casp3 | 0.68 |
|  |  | Il15 | 1.21 |
| RIG-I-like receptor signaling | | Traf3 | 0.64 |
| Irf7 | 5.42 | Ccl12 | 4.19 |
| Dhx58 | 3.33 | Rela | 0.61 |
| Ifih1 | 1.81 |  |  |
| Tmem173 | 1.13 | Toll-like receptor signaling | |
| Cxcl10 | 2.38 | Tlr1 | 1.21 |
| Ikbke | 1.99 | Tlr9 | 2.20 |
| Tradd | 1.03 | Tlr6 | 0.76 |
| Trim25 | 0.82 | Traf3 | 0.64 |
| Traf2 | 0.83 | Ikbke | 1.99 |
| Casp8 | 0.76 | Irf7 | 5.42 |
| Azi2 | 0.72 | Stat1 | 2.20 |
| Nfkbib | 1.03 | Spp1 | 2.37 |
| Traf3 | 0.64 | Cxcl9 | 3.67 |
| Otud5 | 0.45 | Cxcl10 | 2.38 |
| Rela | 0.61 | Cd40 | 1.85 |
|  |  | Il1b | 1.85 |
| NF-kappa B signaling | | Ccl5 | 1.27 |
| Ddx58 | 2.34 | Casp8 | 0.76 |
| Traf1 | 1.51 | Cd80 | 0.66 |
| Ptgs2 | 5.60 | Map2k2 | 0.64 |
| Cd40 | 1.85 | Map2k4 | 0.62 |
| Il1b | 1.85 | Il6 | 0.81 |
| Plau | 2.25 | Cd14 | 0.90 |
| Vcam1 | 0.86 | Ifnar1 | 0.52 |
| Tradd | 1.03 | Irf5 | 0.96 |
| Nfkb2 | 0.85 | Ctsk | 0.67 |
| Trim25 | 0.82 | Rela | 0.61 |
| Traf2 | 0.83 | Map2k1 | 1.22 |
| Pias4 | 0.64 |  |  |
| Relb | 0.78 |  |  |
| Cd14 | 0.90 |  |  |
| Csnk2b | 0.64 |  |  |
| Tnfrsf11a | 0.83 |  |  |
| Traf3 | 0.64 |  |  |
| Rela | 0.61 |  |  |
| Csnk2a2 | 0.44 |  |  |
|  |  |  |  |
| **Down-regulated genes by IAV vs. PBS control** | | | |
| Genes | Log2 fold change | Genes | Log2 fold change |
| Focal adhesion |  | Tight junctions | |
| Itga8 | -2.84 | Tjp1 | -1.74 |
| Tnxb | -1.81 | Nedd4 | -1.21 |
| Lama2 | -2.20 | Patj | -1.12 |
| Cav1 | -1.29 | Myh10 | -1.13 |
| Lamc3 | -2.09 | Afdn | -0.78 |
| Lama5 | -1.20 | Cldn5 | -1.63 |
| Itga1 | -1.09 | Magi1 | -1.33 |
| Col4a3 | -2.38 | Cldn1 | -0.78 |
| Cav2 | -0.96 | Cd1d2 | -5.88 |
| Reln | -1.94 | Jam2 | -1.02 |
| Chad | -2.27 | Marveld2 | -0.87 |
| Ppp1r12b | -0.94 | Myh14 | -0.88 |
| Prkcb | -0.97 | Map3k5 | -0.86 |
| Vwf | -1.05 | Amotl1 | -0.87 |
| Rac3 | -1.32 | Rdx | -0.55 |
| Lamb1 | -1.02 | Prkce | -0.79 |
| Pik3r3 | -1.73 | Pard6g | -1.10 |
| Pdgfrb | -1.04 | Cgnl1 | -0.63 |
| Col4a5 | -1.67 | Rock2 | -0.56 |
| Mylk | -1.14 | Amot | -1.19 |
| Lamb2 | -1.08 | Dlg1 | -0.59 |
| Pdgfd | -1.71 | Amotl2 | -0.83 |
| Col4a4 | -1.80 | Myh11 | -1.19 |
| Col6a3 | -0.95 | Runx1 | -0.57 |
| Vcl | -0.73 | Mpdz | -1.15 |
| Itga11 | -1.09 | Jun | -0.82 |
| Mylk3 | -5.35 | Epb41l4b | -1.02 |
| Lama3 | -1.39 |  |  |
| Vav2 | -0.58 |  |  |
| Col6a2 | -1.04 |  |  |
| Col6a1 | -1.12 |  |  |
| Col4a6 | -1.85 |  |  |
| Rock2 | -0.56 |  |  |
| Flt4 | -0.74 |  |  |
| Fn1 | -0.66 |  |  |
| Lama4 | -0.68 |  |  |
| Tln2 | -1.32 |  |  |
| Prkcg | -1.13 |  |  |
| Hgf | -1.15 |  |  |
| Thbs3 | -0.75 |  |  |
| Jun | -0.82 |  |  |
| Dock1 | -0.88 |  |  |
