# Supplementary material for: Cooperation of immune regulators Tollip and surfactant protein A inhibits influenza A virus infection in mice
Source: Respir Res. 2024 May 3;25:193. doi: 10.1186/s12931-024-02820-3 (PMC11068576; doi:10.1186/s12931-024-02820-3)
Supplement: Supplementary file 14 — Additional file 14: Supplementary Table 3. Pathways of genes altered by Tollip/SP-A deficiency (vs. SP-A deficiency) in mouse lung macrophages infected with IAV. A table of signaling pathways, the gene count, the percent up- or down- regulated, and the p-value of important pathways found to be associated in mouse lung macrophages. [file 12931_2024_2820_MOESM14_ESM.docx]

**Supplementary Table 3.** Pathways of genes altered by Tollip/SP-A deficiency (vs. SP-A deficiency) in mouse lung macrophages infected with IAV

| **Signaling Pathways** | **Gene Count** | **%** | **Up- or Down- Regulated** | **P value** |
| --- | --- | --- | --- | --- |
| NOD-like receptor signaling pathway | 22 | 7.8 | Up | 1.67E-06 |
| Influenza A | 17 | 6.0 | Up | 4.43E-04 |
| T cell receptor signaling pathway | 12 | 4.2 | Up | 1.24E-03 |
| Cytokine-cytokine receptor interaction | 21 | 7.4 | Up | 3.52E-03 |
|  |  |  |  |  |
| ECM-receptor interaction | 20 | 4.8 | Down | 9.64E-08 |
| Cell adhesion molecules (CAMs) | 24 | 5.8 | Down | 1.20E-05 |
| Focal adhesion | 28 | 6.7 | Down | 4.51E-05 |
