# Supplementary material for: Cooperation of immune regulators Tollip and surfactant protein A inhibits influenza A virus infection in mice
Source: Respir Res. 2024 May 3;25:193. doi: 10.1186/s12931-024-02820-3 (PMC11068576; doi:10.1186/s12931-024-02820-3)
Supplement: Supplementary file 15 — Additional file 15: Supplementary Table 4. List of genes in selected pathways altered by Tollip/SP-A deficiency (vs. SP-A deficiency) in mouse lung macrophages infected with IAV. A table of genes and the log2 fold change associated with the pathways listed in Supplementary table 3. [file 12931_2024_2820_MOESM15_ESM.docx]

**Supplementary Table 4.** List of genes in selected pathways altered by Tollip/SP-A deficiency (vs. SP-A deficiency) in mouse lung macrophages infected with IAV

| **Up-regulated genes by IAV vs. PBS control** | | | |
| --- | --- | --- | --- |
| Genes | Log2 fold change | Genes | Log2 fold change |
| Influenza A | | Cytokine-cytokine receptor interaction | |
| Il18 | 1.25 | Il18 | 1.25 |
| Ifng | 1.52 | Ifng | 1.52 |
| Fasl | 1.07 | Cxcl9 | 1.00 |
| Nlrp3 | 0.64 | Il18rap | 0.98 |
| Tlr7 | 0.68 | Xcl1 | 1.28 |
| Ep300 | 0.78 | Il2rb | 0.87 |
| Pik3cd | 0.56 | Il12rb1 | 1.07 |
| Adar | 0.58 | Ccr9 | 1.19 |
| Ccl5 | 0.60 | Ccl5 | 0.60 |
| Ifih1 | 0.50 | Cxcr1 | 0.92 |
| Eif2ak3 | 0.54 | Tnfrsf9 | 0.68 |
| Tnf | 0.52 | Tnf | 0.52 |
| Stat2 | 0.52 | Tgfbr1 | 0.52 |
| Oas3 | 0.48 | Tnfrsf21 | 0.56 |
| Rsad2 | 0.46 | Il16 | 0.57 |
| Crebbp | 0.53 | Clcf1 | 0.52 |
| Jak1 | 0.45 | Cxcr5 | 0.55 |
|  |  | Csf2ra | 0.45 |
|  |  | Csf3r | 0.44 |
|  |  |  |  |
| **Down-regulated genes by IAV vs. PBS control** | | | |
| Genes | Log2 fold change | Genes | Log2 fold change |
| Focal adhesion |  | Cell adhesion molecules (CAMs) | |
| Itga8 | -2.90 | Cldn5 | -1.67 |
| Itga1 | -1.55 | Esam | -1.85 |
| Cav1 | -1.04 | Icam2 | -1.50 |
| Col4a2 | -1.25 | Cdh5 | -1.68 |
| Pdgfra | -1.03 | Itga8 | -2.90 |
| Col6a3 | -1.24 | Pecam1 | -1.06 |
| Col4a1 | -1.16 | Cldn18 | -1.74 |
| Tnc | -1.08 | Cadm1 | -1.23 |
| Mylk | -1.14 | Ptprm | -1.42 |
| Col6a2 | -0.92 | Cdh1 | -0.85 |
| Parva | -0.92 | Jam2 | -1.36 |
| Vwf | -0.94 | Nrcam | -2.08 |
| Myl9 | -1.40 | Nectin3 | -1.24 |
| Itga3 | -0.91 | Cd34 | -0.73 |
| Col6a1 | -0.87 | Cldn3 | -1.05 |
| Bcar1 | -0.83 | Sdc4 | -0.60 |
| Pgf | -0.66 | Sdc2 | -0.91 |
| Pdgfd | -1.16 | Sdc1 | -0.71 |
| Itga7 | -1.12 | Cdh4 | -3.89 |
| Ccnd1 | -0.74 | Cldn7 | -1.13 |
| Col1a2 | -0.53 | Vcam1 | -0.50 |
| Col4a4 | -1.41 | Cdh15 | -5.54 |
| Col1a1 | -0.53 | Itga9 | -0.64 |
| Bad | -0.71 | Ptprf | -0.79 |
| Lama1 | -3.24 |  |  |
| Vegfd | -0.68 |  |  |
| Itga9 | -0.64 |  |  |
| Ptk2 | -0.68 |  |  |
